# Report card on school snack food policies among the United States' largest school districts in 2004–2005: Room for improvement

**DOI:** 10.1186/1479-5868-3-1

**Published:** 2006-01-03

**Authors:** H Mollie Greves, Frederick P Rivara

**Affiliations:** 1 Department of Pediatrics, University of Washington and the Children's Hospital Regional Medical Center, 4800 Sand Point Way NE, Box 359300, G0007 Seattle, WA 98105-0371, USA; 2 Child Health Institute, University of Washington, Box 354920 Seattle WA 98195-4920, USA

## Abstract

**Background:**

Federal nutritional guidelines apply to school foods provided through the national school lunch and breakfast programs, but few federal regulations apply to other foods and drinks sold in schools (labeled "competitive foods"), which are often high in calories, fat and sugar. Competitive food policies among school districts are increasingly viewed as an important modifiable factor in the school nutrition environment, particularly to address rising rates of childhood overweight. Congress passed legislation in 2004 requiring all school districts to develop a Wellness Policy that includes nutrition guidelines for competitive foods starting in 2006–2007. In addition, the Institute of Medicine (IOM) recently published recommendations for schools to address childhood obesity.

**Methods:**

Representatives of school districts with the largest student enrollment in each state and D.C. (N = 51) were interviewed in October-November 2004 about each school district's nutrition policies on "competitive foods." District policies were examined and compared to the Institute of Medicine's recommendations for schools to address childhood obesity. Information about state competitive food policies was accessed via the Internet, and through state and district contacts.

**Results:**

The 51 districts accounted for 5.9 million students, representing 11% of US students. Nineteen of the 51 districts (39%) had competitive food policies beyond state or federal requirements. The majority of these district policies (79%) were adopted since 2002. School district policies varied in scope and requirements. Ten districts (53%) set different standards by grade level. Most district policies had criteria for food and beverage content (74%) and prohibited the sale of soda in all schools (63%); fewer policies restricted portion size of foods (53%) or beverages (47%). Restrictions more often applied to vending machines (95%), cafeteria à la carte (79%), and student stores (79%) than fundraising activities (47%). Most of the policies did not address more comprehensive approaches to the school nutrition environment, such as nutrition education (32%) or advertising to students (26%), nor did they include guidelines on physical education (11%). In addition, few policies addressed monitoring (32%) or consequences for non-compliance (11%). No policy restricted foods sold for after-school fundraising or required monitoring physical health indicators (e.g. BMI).

**Conclusion:**

When compared to the Institute of Medicine's recommendations for schools' role in preventing obesity, none of the nutrition policies among each state's largest school district had addressed all the recommendations by 2004–2005. Nutritionists, nurses, pediatricians, parents, and others concerned about child health have an unprecedented opportunity to help shape and implement more comprehensive school district nutrition policies as part of the Congressional requirement for a "Wellness Policy" by 2006–2007.

## Introduction

Snack foods and beverages high in sugar, fat and calories are now widely available in schools [1-3], and children's intake of these foods has increased [4]. While the United States Department of Agriculture (USDA) regulates the nutritional content of foods sold in the national school lunch and breakfast programs, federal guidelines for foods sold outside these programs (labeled "competitive foods") are limited [5]. When competitive foods are available in schools, children consume more fat and sugar and consume less fruits, vegetables and milk [6-9]. Schools face a dilemma in restricting the sale of snack foods and beverages because they generate significant revenue, especially in exclusive contracts with soda companies [10,11].

Recognizing that unhealthy foods and beverages in schools may contribute to growing problems of childhood obesity and poor nutrition, the USDA reported to Congress in 2001 on the magnitude of the problem of competitive food sales in schools [12]. Since then, multiple efforts have been initiated and expanded at the federal, state and local levels to decrease unhealthy competitive foods in the school setting [13-18]. National agencies, including the Centers for Disease Control and Prevention (CDC) [16], the American Academy of Pediatrics (AAP) [19], and the Institute of Medicine (IOM) [20], have all published recommended changes to the school nutrition environment, particularly competitive foods, as part of efforts to address childhood overweight.

In 2004, Congress established a requirement for all school districts to adopt a "Wellness Policy" including nutrition guidelines for all foods available on school campuses during the school day, as well as guidelines for nutrition education and physical activity [21]. Prior to this Congressional act, many states and school districts had already initiated policy changes to extend nutritional guidelines to competitive foods [22-24]. Although reports and studies on local or regional school nutrition policies for competitive foods are available [25-27], no analysis of school district nutrition policies across the country had been published since the 2000 School Health Policies and Programs Study (SHPPS) [28].

We sought to examine nutrition policies for competitive foods in the largest school districts in each state for 2004–2005 and to compare these to published recommendations of the Institute of Medicine (See Table 1). This study highlights the magnitude of change required among the largest school districts across the country as districts move forward to comply with adopting Congressionally-mandated Wellness Policies by 2006–2007. This study demonstrates the need for ongoing involvement of nutritionists, nurses, pediatricians, parents, and others concerned about child health to help develop and implement more comprehensive school district nutrition policies.

**Table 1:** Summary of Institute of Medicine Recommendations for Schools to Address Childhood Obesity, September 2004

■ Establish nutritional standards for all "Competitive Foods"
■ Establish a minimum of 30 minutes of activity during school day
■ Enhance school health curricula
■ Ensure all school meals meet the Dietary Guidelines for Americans
■ Ensure schools are as advertising free as possible
■ Conduct annual assessments of student weight, height, BMI, and make data available to parents

## Methods

One investigator (M.G.) made contact by phone and email in October-November 2004 to representatives of the school districts with the largest enrollment in each state and the District of Columbia. We surveyed a district from each state to capture a snapshot of district policies across the country in which different state laws apply. We chose districts with the largest student enrollments because their policies affect the most children in each state. In addition, the largest districts, representing primarily urban settings, enroll a larger proportion of minority students [29], who are disproportionately affected by problems of overweight [30], live in communities with less access to fresh fruits and vegetables [25], and have been found to consume more fast foods [31].

Information was collected in three domains: 1) demographic characteristics of the student body, 2) district nutrition policies on competitive foods and beverages, and 3) current competitive food environment including district contracts with soda vendors. Whenever available, information about nutrition policies was first obtained from each school district's website and then confirmed with personal communications. In most districts, the director of Food/Nutrition Services, or a district dietician or nutritionist serving as a spokesperson for the director, provided this information. In cases when the director of food service or their representatives were uncertain of the district nutrition policy, in particular for vending contracts that are often outside the domain of Food Services, follow-up contact was made with district personnel managing vending contracts.

Copies of all written policies on nutrition policies for competitive foods were requested and reviewed and were compared with data provided by personal communications. Demographic information was primarily collected from the most recent publicly published data available on the school district's website, or from the district's office of public information.

We defined a school district "competitive food policy" as any policy beyond state or federal guidelines that consistently applied to foods and/or beverages sold outside the school lunch or breakfast program in one or more of the following settings: vending machines, cafeteria à la carte, school stores, and fundraising activities. We included policies passed by the school board, the district superintendent, or those implemented by district departments of food/nutrition services. We included *de facto *policies that were not written only if the policy universally applied to competitive foods sold in the school district in one or more of the above settings. Competitive food policies at the state level were assessed separately in order to identify district policies that extended beyond state policies.

Nutrition policies on competitive foods were compared on 1) restrictions on content (i.e. sugar, fat, and/or sodium), 2) restrictions on size of food and beverages sold, 3) venues where policy applies (i.e. vending, cafeteria à la carte, school stores, fundraising), 4) time of day when policy applies, 5) differences in policies by grade level, 6) school accountability for nutrition policies, and 6) if the policy addresses other areas of wellness as recommended by the Institute of Medicine: nutrition education, food advertising/marketing to students, reporting of body mass index (BMI), or guidelines for physical activity [20].

In addition to assessing particular school district policies, we also collected data about the current nutrition environment for competitive foods in all 51 school districts. These data included whether soda vending was allowed in the elementary, middle or high schools, whether branded fast foods are sold in the schools that do not meet USDA nutrition guidelines, and whether they have an exclusive district soda vending contract.

Finally, in order to compare district policies in the context of different state requirements, we reviewed state competitive food policies. These were obtained from state websites and via state and district personnel.

## Results

The 51 school districts assessed in this study represent approximately 5.9 million students enrolled in 8,310 schools across the US (Table 2). Combined, these school districts account for 11% of the 53 million students in the U.S. and 6% of the 129,000 schools [32]. Enrollment in these school districts ranges from 3,500 in Burlington, VT to 1,086,886 in New York City. Student eligibility for free and reduced school lunch in this sample of school districts ranged from 14% to 88%. Over half of the districts (N = 28) had greater than 50% of students eligible for free and reduced lunches.

**Table 2:** Demographic characteristics of largest school district in each state and District of Columbia with (N = 19) and without (N = 32) competitive food policy more restrictive than state and federal policy in 2004–2005

	Total all districts (N = 51)	Districts without policies (N = 32)	Districts with policies (N = 19)
Total enrollment	5,924,261	2,105,903	3,8182,358
Mean	116,162	65,809	200,966
Median	60,300	47,179	98,000
Range	3,500–1,086,886	13,000–211,499	3,500–1,086,886
Number of schools	8,310	3,403	4,907
Mean # per district	163	106	258
Median # per district	103	93	167
Range	10–1,330	32–307	10–1,330
Percent range qualifying for free/reduced lunch	14–88%	18%–84%	20–88%
Districts with >50% students qualifying for free/reduced lunch	28 (55%)	18 (56%)	10 (53%)

### District competitive food policies

Nineteen (39%) of the 51 school districts surveyed had voluntarily adopted a nutrition policy for competitive foods beyond state or federal requirements. School districts that had adopted competitive food policies had larger mean and median enrollments than the 32 districts without such policies (Table 2). Among districts with competitive food policies, a similar proportion had greater than 50% eligibility for free and reduced lunches: 10 of 19 (53%) with policies, versus 18 of 32 (56%) without policies.

The nutrition policies for competitive foods adopted by the 19 school districts varied in scope and requirements (Tables 3 and 4). Fifteen of the 19 districts (79%) had new or revised policies since 2002. Only one district adopted a "guidelines only" policy, but in practice, the district had implemented specific nutrition guidelines for most foods sold in the district. About half of the districts (53%) set different standards by grade level, with more restrictive policies for elementary schools and more permissive policies for secondary schools. Twelve of 19 school district policies (63%) prohibited the sale of soda in *all *schools.

**Table 3:** Competitive food policies of the largest school district in each state & D.C. for 2004–2005 (districts with policy more restrictive than state and federal policies, N = 19)

**Policy Components**	**Number**	**Percent (N = 19)**
***Time of day and venue where policy applies***		
General recommendations only	1	5%
Specifically applies only to foods/ beverages sold during the school day	9	47%
Applies to vending machines	18	95%
Applies to cafeteria à la carte	15	79%
Applies to student stores	15	79%
Applies to fundraising activities	9	47%
Applies to after-school fundraising or concessions	0	--
Different standards set for grade levels	10	53%
***Content and portion size***		
Restricts food content	14	74%
Restricts food portion size	10	53%
Restricts sugar content of juice drinks	14	74%
Restricts portion size of beverages	9	47%
Restricts soda in all schools	12	63%
***Policy elements extending beyond competitive foods***		
Addresses marketing to students	5	26%
Addresses nutrition education	6	32%
Addresses physical education	2	11%
Addresses monitoring/compliance procedures	6	32%
Includes consequences for non-compliance	2	11%
Addresses measure of physical health (i.e. BMI)	0	--

**Table 4:** Comparison of competitive food policies (beyond state or federal guidelines) of largest school districts in each state and the District of Columbia (2004–2005)

									Policy Includes Clause to Address				
State	School District	Policy limited to school hours	Stds differ by grade level	Restricts food content	Restricts food portions	Restricts % sugar in juice drinks	Restricts drink portion	No sodas	Marketing	Nutr. Edu.	PE	Monitoring	Non- Compliance

CA	Los Angeles Unified												
DE	Christina												
--	District of Columbia												
FL	Dade Co.												
IL	Chicago												
KY	Jefferson Co.												
ME	Portland												
MA	Boston												
NV	Clark Co.												
NH	Manchester												
NY	NYC												
ND	Grand Forks												
OR	Portland												
PA	Philadelphia												
TN	Memphis City Schools												
VT	Burlington												
VA	Fairfax Co.												
WA	Seattle												
WI	Milwaukee												
	Total	9	10	14	10	14	9	12	5	6	2	6	2
	Percent (n = 19)	47%	53%	74%	53%	74%	47%	63%	26%	32%	11%	32%	11%

District policies restricted content (e.g. fat, sugar, and sodium) more frequently than portion size for both food and beverages. Almost three-quarters of policies established nutritional criteria for content limiting fat, sugar, and sodium in foods approved for sale, although some districts had limited those requirements to a certain percentage of the foods offered (e.g. 60% of vending machine items must be healthy). Ten of 19 policies (53%) limited the portion size of food items and 9 (47%) limited the size of beverages.

Competitive food policies did not typically apply to all settings in which food is sold in schools. Many of the school districts had adopted policies for foods and beverages sold in vending machines, but not for other venues. All but one of the policies (95%) applied to foods and beverages sold in vending machines, but less than half (47%) applied to items sold for school fundraising activities. Almost half of school districts (47%) specifically limited the policy only to foods sold during the school day. None of the school districts included after-school fundraising or concession sales as part of their policies.

A limited number of the districts had extended nutrition policies beyond competitive foods in 2004–2005 to create more comprehensive "wellness" policies, such as nutrition education (26%), guidelines for advertising or marketing to students (26%), and physical activity (11%). None of the policies required objective measures of student health or nutrition status such as measuring BMI. Six of the 19 policies (32%) mentioned monitoring and compliance, but only two (10%) included consequences for non-compliance.

### District sale of soda and branded products

In addition to assessing competitive food policies, we surveyed current district practices for the competitive food environment regarding soda sales, branded fast foods, and district-wide contracts with beverage vendors (Table 5). Twelve school districts (24%) prohibited soda sales in all schools. Among the 51 districts in this sample, only 14% permitted soda vending in elementary schools, but the majority allowed soda vending in middle (61%) and high schools (75%). Three school districts (6%) were using branded fast food vendors (e.g. Pizza Hut, Taco Bell, McDonald's etc) in à la carte menus without requiring those foods meet USDA dietary requirements. Many more districts (41%) were selling branded fast foods like pizza as part of the school lunch, made to specification to meet USDA lunch guidelines. Fifteen school districts (29%) of the 51 total had exclusive contracts with a beverage vendor. A similar percentage of districts with and without competitive food policies had exclusive beverage contracts (32% versus 28%).

**Table 5:** Competitive food environment (2004–2005) in the largest school district in each state & D.C.

District competitive food restrictions and allowances	Number	Percent (N = 51)
School districts that *prohibit *soda sales at all schools	12	24%
Districts that *allow *soda vending in elementary schools	7	14%
Districts that *allow *soda vending in middle schools	31	61%
Districts that *allow *soda vending in high schools	38	75%
School districts using branded fast food vendors in à la carte sales that do *not * meet USDA nutritional guidelines for school lunch (such as Pizza Hut, Taco Bell, and/or McDonald's)	3	6%
District-wide exclusive contracts for beverage vending	15	29%

### State competitive food policies

Prior to 2002 when the USDA summarized state policies for competitive foods [24], 20 states had state legislation regulating sales of competitive foods beyond those required by federal regulations (Table 6). Eleven states had passed policies (either legislated or required by state agencies) that addressed competitive foods from 2002–2004. Seven of these 11 states *required *stricter limits than federal regulations on competitive foods sold in the schools. Four of the 11 states made *recommendations *for changes to competitive food policies, primarily by requiring state agencies or committees to develop model policies. The most comprehensive state-mandated competitive food policies were in California, Hawaii, Texas and West Virginia. Each of these states adopted requirements for schools to offer only competitive foods that meet certain nutritional guidelines, although more restrictive requirements were limited to elementary schools, with more permissive policies for secondary schools (Table 7).

**Table 6:** State competitive food policies summary

State competitive food policies	Total Number (States)	Percent (N = 50)
States with legislation more restrictive than federal requirements regarding competitive food sales in schools prior to 2002	20	40%
States with new competitive food policies that *require *or *will require *changes passed since 2002	7 (Arkansas, California*, Connecticut**, Hawaii*, Texas*, Tennessee, West Virginia*)	14%
States with new policies that *recommend *changes to competitive food sales passed since 2002	4 (Colorado, North Carolina, Vermont, Washington)	8%
States with legislation to address childhood obesity, by nutrition and/or physical activity passed since 2002	16 (Arkansas, California, Colorado, Connecticut, Florida, Louisiana, Mississippi, Nevada, New Mexico, North Carolina, Oklahoma, Texas, Tennessee, Vermont, Washington, West Virginia)	32%

* Requires schools to offer only competitive foods that meet certain nutritional guidelines

**Requires certain healthy foods be sold whenever sodas/fruit drinks are available

**Table 7:** State legislation and policies passed since 2002 regarding childhood obesity: competitive foods, nutrition, and physical activity

State	Revised competitive food policy	Policy req'ts vary by grade level	Model policy for comp. foods	State "wellness policy"	Nutrition education	PE/Physical activity	Measure BMI	Advisory committee(s)
AR								
CA*								
CO	*R*							
CT								
FL					*R*	*R*		
HI*								
LA							*P*	
MI								
NV						*R*		
NM								
OK								
TN								
TX*								
VT					*R*	*R*	*R*	
WA								
WV*								

*Denotes states that have specific guidelines for nutritional content of competitive foods

*R *= recommendation

*P *= pilot program

## Discussion

The Committee on evaluation of Children's Health of the National Research Council and Institute of Medicine have recently proposed a new conceptual model for children's health. In this model, the biological, behavioral and social and physical environmental influences on children's health operate within the broader context of policy and services. The food policies in schools are an important part of the broader context which affects children's health around obesity and physical activity [33].

In this survey of competitive food policies among the largest school districts in each state and the District of Columbia, we found that substantial changes to nutrition policies and foods offered at schools had occurred by 2004–2005. None of the districts, however, had adopted a policy that met all recommendations of the Institute of Medicine guidelines for the role of schools in preventing childhood obesity [20]. Overall, the Los Angeles Unified School District, which passed the first new competitive food policy among the largest districts in the U.S., had the most comprehensive policy.

The majority of school district policies that had been adopted since 2002 sought to impact the type and quantity of competitive foods and beverages available by setting specific limits on content and portions. Portion sizes of foods were more often restricted than those of beverages.

Almost universally, the representatives of nutrition services interviewed described multiple changes in recent years to improve nutrition in the schools, such as offering more fresh fruits and vegetables and eliminating regular chips, fried foods, and sodas from the cafeteria à la carte menus. Assessing changes to the school lunch program was beyond the scope of this study, however many school districts noted implementation of part or all of the USDA Healthy School Meals Initiative [34] and the CDC Coordinated School Health Program Guidelines for School Health Programs to Promote Lifelong Healthy Eating [17]. Based on these findings, we predict multiple positive changes in the next version of the School Health Policies and Programs Study (SHPPS), scheduled for 2006 [28].

School nutrition representatives cited the financial impact of limiting competitive foods as the major obstacle among school districts in adopting a competitive foods policy, specifically one that limits the sale of sodas. Anecdotal reports from some schools and districts show no detrimental financial impact in converting to healthy vending options [35]. However, no published studies have examined this issue. Contrary to our expected findings, school districts with exclusive vending contracts were not less likely to have adopted a competitive foods policy. Both districts with exclusive vendor contracts (e.g. Coca-Cola or Pepsi), and those with individual school contracts, grappled with anticipated financial losses if soda sales were restricted. In fact, in school districts without a district-wide contract, nutrition services personnel frequently cited resistance from individual school principals in developing a policy restricting soda sales. Several school districts adopted new district-wide vending contracts in order to centralize purchasing and approval of foods and beverages sold. Many were placing the management of new contracts under the division of food or nutrition services in order to ensure better nutritional content of vended items.

Beyond the financial constraints, respondents identified several additional barriers to adopting and implementing a competitive food policy. One barrier was the lack of priority among school district administrators to address child nutrition. Respondents from nutrition services in some districts described their struggle to find support among administrators or school board members to champion the cause for improving nutrition, particularly given the burden of increasing requirements for achieving academic benchmarks. Another barrier in some districts were parents and students who resisted changes to the school's food and drink offerings, wanting to protect students' "free will" in choosing what they eat, even if it is unhealthy. These aforementioned barriers are likely to remain an issue for school districts as they move forward to adopt and implement Wellness Policies.

Successful implementation of new competitive foods policies in individual schools across the districts is relatively unknown. Nutrition services personnel often noted school non-compliance under current USDA requirements to keep vending machines off during mealtimes, depending on individual school administrators' oversight and commitment to compliance. In this study, less than one-third of the competitive food policies included clauses for monitoring and enforcement, and only two policies included consequences for non-compliance. Based on these findings, individual school compliance with the policies may depend heavily on the advocacy or support by each schools' administrators and staff, and may therefore vary widely from school to school. For those school districts that had opted to require time limitations for sale of competitive foods without content or portion size requirements, non-compliance could be a significant problem, and such policies would likely have little effect.

Current data about the potential impact of these new policy changes is limited. There is evidence that specific foods, especially soft drinks, may contribute to obesity [36,37]. The degree to which eliminating these products from schools will directly impact rates of childhood obesity is uncertain, however. There is agreement among most national organizations about the elements of school nutrition policies that are likely to have an impact, and specific policy guidelines have been recommended by the National Alliance for Nutrition and Activity (NANA) [38], but no optimal school policy to reduce childhood obesity has been identified or tested. Nevertheless, the improved nutrition environment in which children are more likely to consume healthier foods should provide sufficient reason for enacting policies to improve school nutrition. A promising outcome of policy changes reported by district representatives in this study was to note that snack food and beverage vendors were changing product lines and developing new products in order to comply with new district and state policies.

Limitations of this study include possible incomplete or inaccurate information provided by nutrition and food services representatives. Every reasonable effort was made to verify information when nutrition personnel were uncertain about the district's policies or plans, particularly for vending, which is often managed by the purchasing department. Another study limitation is that changes seen in the larger urban school districts may not be generalizable to smaller school districts. Our sample included a few smaller districts in less populated states, which reported similar rates of new policies and faced similar challenges in incorporating policies. Finally, these data represent a summary of the largest districts for 2004–2005 school year and may not be current as policies and state legislation are changing continually.

In our study of nutrition policies for competitive foods in the largest school districts across the country in 2004–2005, we found more than one-third of districts had adopted new competitive food policies since 2002. Most policies required specific criteria for sales of healthier foods and beverages. For example, over 60% of policies prohibited sales of soda in all schools, which based on available data, may have the most potential for impacting childhood obesity [36,37]. At the state level, eleven states had adopted new policies for competitive foods in schools, but only four of these had specific school nutrition guidelines.

None of the school district policies in 2004–2005 among states or their largest school districts met the Institute of Medicine's recommendations for schools' role in preventing obesity. Notable gaps in nutrition policies included not establishing portion sizes for both foods and beverages, not addressing fundraising or marketing food to students, and not measuring physical health indicators. In addition, most policies in 2004–2005 did not include guidelines for nutrition education or physical activity. Finally, few policies addressed monitoring or consequences for non-compliance.

The USDA is requiring all school districts participating in the National School Lunch Program to develop a Wellness Policy by 2006–2007, including nutrition guidelines for all foods available at school, and guidelines for physical activity and nutrition education [21]. Based on the findings from this study, few large school districts across the country had heretofore adopted a comprehensive Wellness Policy. The requirement to develop a Wellness Policy represents a crucial and unprecedented opportunity for nutritionists, pediatricians, nurses, parents and others interested in child health, to influence school nutrition policies. As school districts adopt new Wellness Policies, studies are needed to assess outcomes, particularly impact on child well-being such as child nutrition and overweight, and financial impact for school districts and their nutrition services departments.
